# TonEBP regulates the hyperosmotic expression of aquaporin 1 and 5 in the intervertebral disc

**DOI:** 10.1038/s41598-021-81838-9

**Published:** 2021-02-04

**Authors:** J. W. Snuggs, S. Tessier, R. A. B. Bunning, I. M. Shapiro, M. V. Risbud, C. L. Le Maitre

**Affiliations:** 1 Biomolecular Sciences Research Centre, Sheffield Hallam University, City Campus, Howard Street, Sheffield, S1 1WB UK; 2 Department of Orthopaedic Surgery, Sidney Kimmel Medical College, Thomas Jefferson University, Philadelphia, PA USA; 3 Graduate Program in Cell Biology and Regenerative Medicine, Thomas Jefferson University, Philadelphia, PA USA

**Keywords:** Mechanisms of disease, Cell biology, Molecular biology, Rheumatic diseases

## Abstract

The central region of the intervertebral disc (IVD) is rich in proteoglycans, leading to a hyperosmotic environment, which fluctuates with daily loading. The cells of the nucleus pulposus (NP cells) have adapted to this environment via the function of tonicity enhancer binding protein (TonEBP), and NP cells have been shown to express several water channels known as aquaporins (AQP). We have previously shown that AQP1 and 5 decrease during IVD degeneration. Here, the regulation of AQP1 and 5 by hyperosmotic conditions and the role of TonEBP in this regulation was investigated. AQP1 and 5 gene expression was upregulated by hyperosmotic conditions mimicking the osmolality of the healthy IVD, which was abrogated by TonEBP knockdown. Furthermore, AQP1 and 5 immunopositivity was significantly reduced in TonEBP^Δ/Δ^ E17.5 mice when compared with wildtype controls, indicating in vivo expression of AQP1 and 5 is controlled at least in part by TonEBP. This hyperosmotic regulation of AQP1 and 5 could help to explain the decreased AQP1 and 5 expression during degeneration, when the osmolality of the NP decreases. Together this data suggests that TonEBP-regulated osmo-adaptation may be disrupted during IVD degeneration when the expression of both AQPs is reduced.

## Introduction

Many factors contribute to the function of the nucleus pulposus (NP). The environment in which NP cells reside plays a role in the physiology of the tissue; biomechanical loading, pH, nutrition, O_2_ tension and adaptation to a diurnal cycle all allow NP tissues to function correctly^1–5^. High content of negatively charged, hydrophilic glycosaminoglycans (GAGs) within the NP matrix allows water and cation retention and provides a high osmotic pressure environment within the disc^1,6,7^. This physiologically hyperosmotic environment has been shown to stimulate matrix synthesis^6,8,9^, providing evidence that NP cells have adapted to the higher osmolality and hydrostatic pressure imposed on them by the NP tissue environment. However, osmotic stress (both hyper- and hypo-) is known to cause many disruptions to cellular activity such as elevation of reactive oxygen species, cytoskeletal rearrangement, inhibition of transcription and translation, damage to DNA and proteins and eventually cell death^10^. Thus, it is important to understand how NP cells have been able to adapt to this constantly changing hyperosmotic environment.

Whilst there are many cell signalling pathways activated during osmotic stress, the role of tonicity enhancer binding protein (TonEBP) (also known as nuclear factor of activated T-cells 5; NFAT5) is argubly the most well-known^11^. Within many tissues TonEBP enables adaptation to a hyperosmotic environment. Whilst the exact mechanisms by which cells sense osmotic shifts is unknown, studies suggest that integrins α_6_β_4_^12^ and α_1_β_1_^13^, a guanine nucleotide exchange factor, Brx^14^, and biomechanical stretching^15^ may all activate TonEBP signalling. When cells are exposed to hyperosmotic stimuli, TonEBP is translocated into the nucleus. In the nucleus it forms homodimers and binds to tonicity response elements (TonE) on target genes, via rel homology domains, causing their upregulation^16^. These well-described target genes include sodium/myo-inositol transporters (SMIT), aldose reductase (AR), betaine-GABA transporter 1 (BGT1), heat shock protein-70 (HSP-70) and taurine transporter (TauT), that all facilitate the exchange of charged ions within cells for small non-ionic osmolytes^7,10,16,17^. Thus, the osmotic pressure across the cell membrane can be restored and cellular adaptation to the hyperosmotic extracellular environment enabled.

TonEBP has an emerging role in the function of the IVD and similar tissues. Under hyperosmotic conditions TonEBP regulates the expression of osmotic response genes^18,19^, aggrecan expression^18^ and key enzyme Beta-1,3-Glucuronyltransferase 3 (β3gat3) involved in synthesis of chondroitin and heparan sulphate sidechains^20^ in NP cells. More recently the function of TonEBP has been shown to be vital during fundamental processes within the spine, as TonEBP deficiency caused delayed notochord and IVD embryogenesis^21^ and an acceleration of age-related IVD degeneration in mice^22^. Whilst in the chondrogenic ATDC5 cell line, TonEBP is involved in the hyperosmotic induction of sox9, collagen II and X, Runx2 and aggrecan^23^. As TonEBP allows NP cells to adapt to their hyperosmotic surroundings, regulate the matrix composition and osmotic status of the NP, it is important to determine what other pathways are potentially regulated by TonEBP to enable the correct function of the IVD.

As the IVD experiences fluctuations in the local osmolality there are potentially many proteins and mechanisms that contribute to the osmoadaptation of the resident cells^24^. One such family of proteins that may contribute are aquaporins (AQP). AQPs are transmembrane channel proteins responsible for the rapid, selective movement of water and other small molecules. These channels contribute to many cellular processes such as cell volume regulation^25^, cell structure and adhesion^26^, cell migration and proliferation^27^ and the overall maintenance of water homeostasis within many tissues^28^.

Many AQPs are expressed within NP tissue^29–31^. Their presence suggests that water transport is tightly controlled and very important for NP cell functionality. The basal expression levels of AQP1 and 5 in NP cells is regulated by HIF-1α^30^, which does not explain why the expression of both AQPs is decreased during IVD degeneration^30^; therefore, other degenerative changes may be implicated in the dysregulation of AQP1 and 5. AQP expression has been shown to be regulated by alterations in extracellular osmolality in many tissues^32–38^. However, only one study to date has investigated this within the context of IVD, demonstrating that TonEBP controlled AQP2 expression under hyperosmotic conditions^39^.

Within other cells/tissues, AQP1 expression is upregulated by hyperosmolality in mIMCD-3 kidney cells^32–34^ and mouse cardiac endothelial cells^35^. AQP5 expression has also been shown to be upregulated by hyperosmolality in 3AO ovarian cancer cells^36^, rat alveolar epithelial cells^37^ and human retinal epithelial cells^38^, in which AQP5 expression was reduced after hypo-osmotic treatment^38^. TonEBP has also been implicated with the hyperosmotic upregulation of both AQP1^33,34^ and AQP5^38^. This potentially indicates that both AQPs are also regulated in the same manner in NP cells, as the osmolality of NP tissue decreases during degeneration. There is also a precedence of AQP regulation in the IVD; AQP3 expression was upregulated by hyperosmolality in murine notochordal (NC) cells^40^ and the hyperosmotic upregulation of AQP2 in rodent NP cells was dependent on TonEBP^39^. If AQP1 and 5 are regulated in this manner, it may implicate them in enabling the adaptation of NP cells to their hyperosmotic environment along with other TonEBP target genes. This adaptation may be diminished during degeneration when their expression is reduced, possibly by the lowered extracellular osmolality.

This study investigated the potential regulation of AQP1 and 5 expression under osmotic conditions representative of the normal and degenerative IVD to identify potential mechanisms that may account for the decreased expression of AQP1 and 5 observed during disc degeneration^30^. Furthermore, the role of TonEBP in the regulation of AQP expression was investigated in IVD cells.

## Results

### Hyperosmotic regulation of AQP1 and 5 gene expression in human NP cells

**Figure 1:**
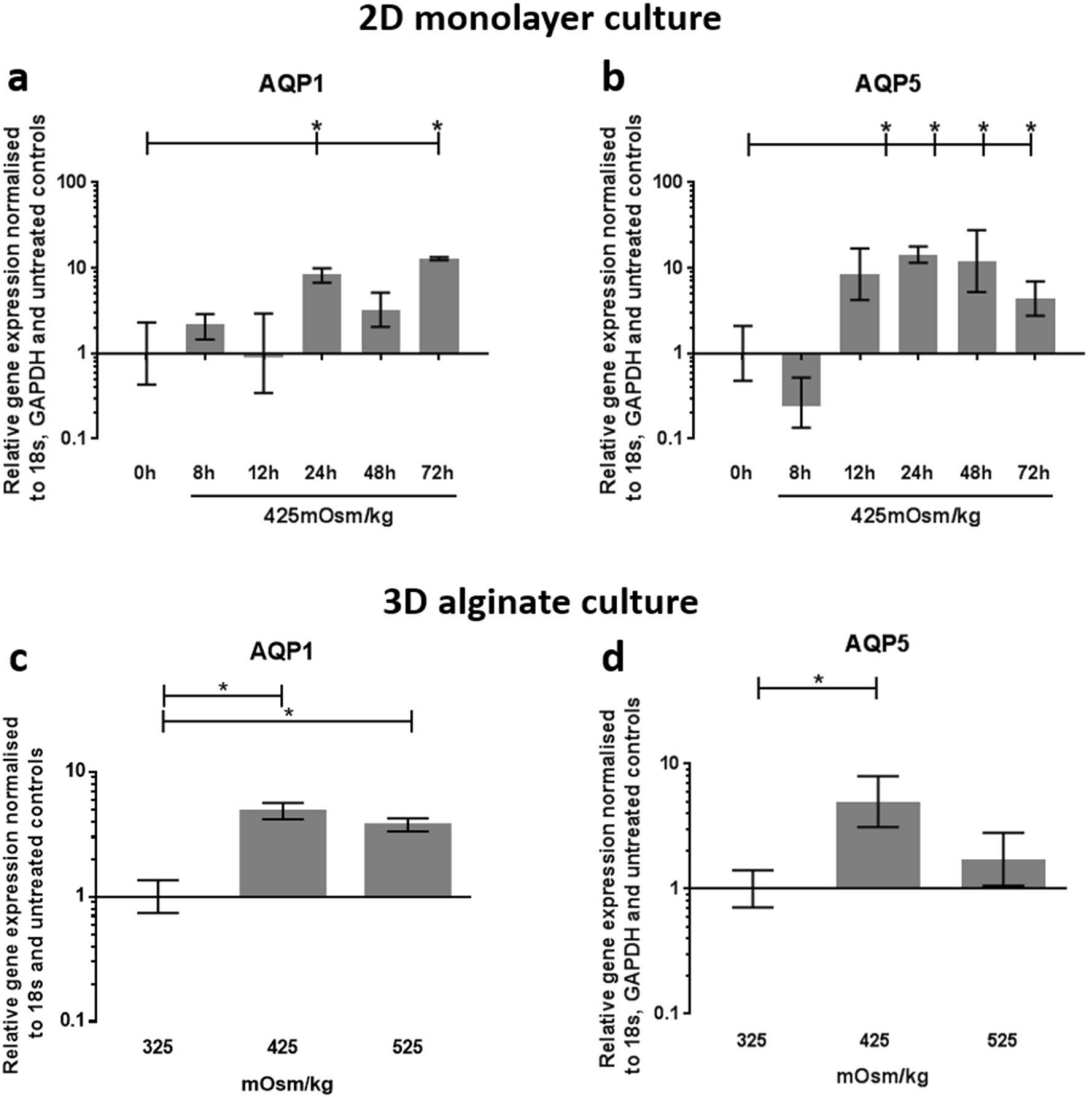
Hyperosmotic regulation of AQP1 and 5 gene expression in human NP cells. Regulation of (**a**) AQP1 and (**b**) AQP5 gene expression, after 8—72 h treatment with 425 mOsm/kg media, in human NP cells cultured in monolayer up to passage 2 prior to treatment. Regulation of (**c**) AQP1 and (**d**) AQP5 gene expression, after 48 h treatment with 425 or 525 mOsm/kg media, in human NP cells cultured for 2w encapsulated in alginate beads prior to treatment. The osmolality of untreated controls in standard culture media was 325 mOsm/kg. The osmolality of treatment media was altered using NaCl and was measured using a freezing point depression osmometer (Model 3320 osmometer, Advanced Instruments). Human NP cells from 3 patients in technical triplicates were utilised for gene expression experiments. Statistical significance determined using Kruskal–Wallis test * = *p* ≤ 0.05.

AQP1 gene expression was significantly upregulated in 2D monolayer cultured human NP cells after 24 h (*p* = 0.046) and 72 h (*p* = 0.005) hyperosmotic treatment (425 mOsm/kg) when compared to untreated controls (325 mOsm/kg) (Fig. 1a). The gene expression of AQP5 in 2D monolayer cultured human NP cells was upregulated by the same hyperosmotic treatment at 12 (*p* = 0.04), 24 (*p* = 0.0041), 48 (*p* = 0.0146) and 72 h (*p* = 0.0342), when compared with untreated controls (Fig. 1b). During 3D alginate culture of human NP cells, which restores the in vivo phenotype, AQP1 gene expression was upregulated by 48 h of 425 mOsm/kg (*p* ≤ 0.0366) and 525 mOsm/kg (*p* = 0.0407) treatment compared to 325 mOsm/kg controls (Fig. 1c), whereas AQP5 expression was only upregulated by 425 mOsm/kg treatment for 48 h, compared to untreated controls (*p* ≤ 0.0271) (Fig. 1d).

### Hyperosmotic regulation of AQP1 and 5 gene expression in rat NP cells

**Figure 2:**
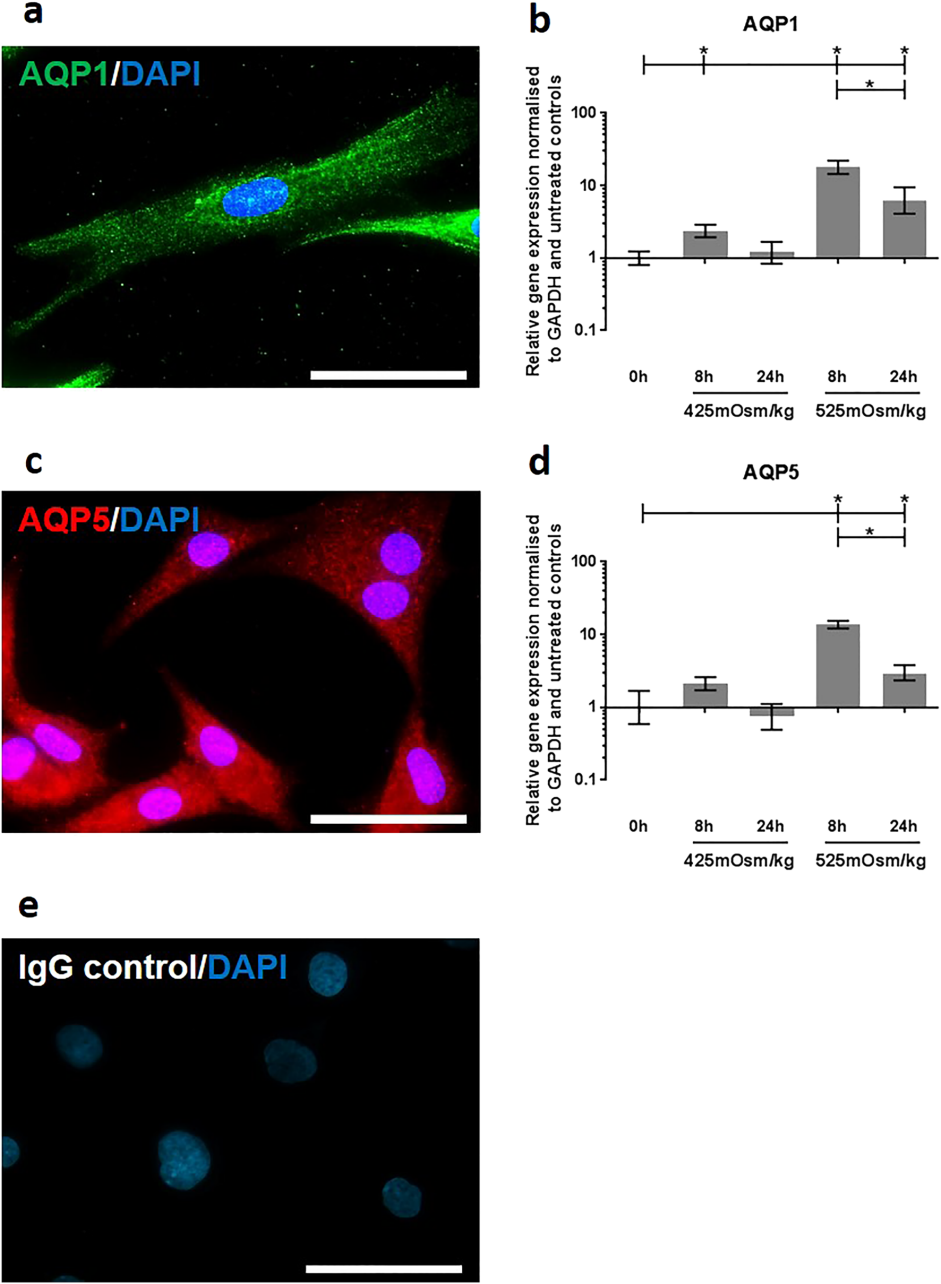
Hyperosmotic localisation of (**a**) AQP1 and (**b**) AQP5 in monolayer cultured rat NP cells. Gene regulation of (**c**) AQP1 and (**d**) AQP5 under hyperosmotic conditions. (**e**) Rabbit IgG isotype control. Scale bar 20 µM. Three repeats using pooled NP cells from 3 rats were utilised for gene expression experiments. Statistical significance determined using Kruskal–Wallis test * = *p* ≤ 0.05.

AQP1 protein in rat NP cells cultured in monolayer was observed by immunofluorescence staining (Fig. 2a). AQP1 gene expression was significantly upregulated in rat NP cells after 8 h treatment with 425 mOsm/kg media (*p* = 0.04) and after 8 h (*p* = 0.021) and 24 h (*p* = 0.03) treatment with 525 mOsm/kg media (Fig. 2b). After 8 h of 525 mOsm/kg media treatment AQP1 gene expression was significantly higher compared to cells exposed to the same treatment for 24 h (*p* = 0.04) (Fig. 2b). Treatment with 425 mOsm/kg media failed to significantly regulate AQP1 gene expression (Fig. 2b). AQP5 protein in rat NP cells in monolayer was assessed by immunofluorescence staining (Fig. 2c). AQP5 gene expression was significantly upregulated in rat NP cells following 8 h (*p* = 0.025) and 24 h (*p* = 0.04) treatment with 525 mOsm/kg media (Fig. 2c). Similar to AQP1 gene regulation, AQP5 gene expression was significantly increased after 8 h of 525 mOsm/kg treatment when compared to 24 h of the same treatment in rat NP cells (*p* = 0.033) (Fig. 2d).

### The effect of TonEBP knockdown on the hyperosmotic regulation of AQP1 and 5 in rat NP cells

**Figure 3:**
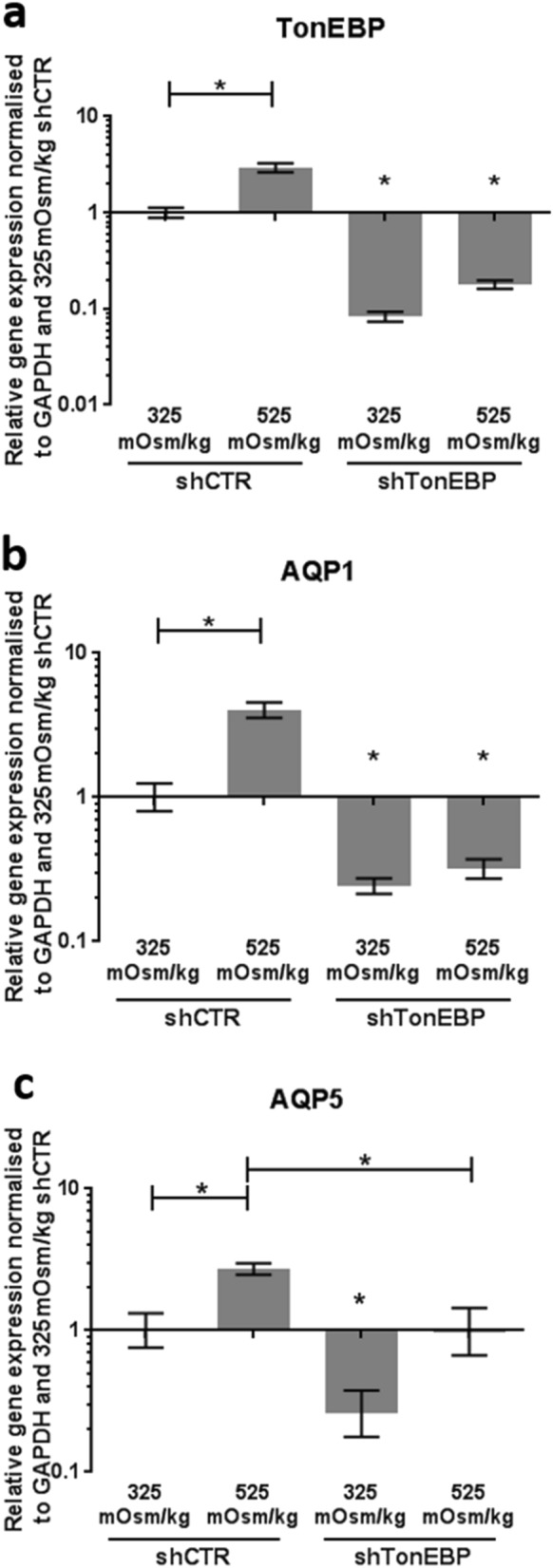
The effects of TonEBP knockdown on the hyperosmotic regulation of AQP1 and 5 in rat NP cells. Rat NP cells were exposed to control (325 mOsm/kg) or hyperosmotic (525 mOsm/kg) treatment for 24 hr, after 4d of TonEBP knockdown (shTonEBP) or no knockdown control (shCTR). After knockdown and treatment, the gene expression of TonEBP (**a**), AQP1 (**b**) and AQP5 (**c**) was determined, respectively. Results were normalised to 325 mOsm/kg shCTR controls. Three repeats using pooled NP cells from 3 rats were utilised for gene expression experiments. Statistical significance determined using Kruskal–Wallis test * = *p* ≤ 0.05.

In rat NP cells transduced with shTonEBP lentiviral vector the gene expression of TonEBP was significantly reduced compared to TonEBP expression in rat NP cells transduced with shCTR (*p* < 0.0001) (Fig. 3a). In shCTR cells, TonEBP gene expression was significantly upregulated in cells treated with 525 mOsm/kg media when compared with untreated controls (325 mOsm/kg) (*p* = 0.021) (Fig. 3a). These results highlight that TonEBP is functional within shCTR cells and expression is successfully knocked down in shTonEBP cells. AQP1 gene expression was upregulated when rat NP cells were treated for 24 h with hyperosmotic media (525 mOsm/kg) in shCTR NP cells (*p* ≤ 0.021) (Fig. 3b). When TonEBP expression was knocked down, AQP1 gene expression in both untreated (*p* < 0.0001) and hyperosmotic (*p* = 0.0003) treatment groups was significantly reduced compared to shCTR controls (Fig. 3b). AQP5 gene expression was still upregulated when rat NP cells were treated for 24 h with hyperosmotic media (525 mOsm/kg) in shCTR NP cells (*p* = 0.0361) (Fig. 3b). When TonEBP expression was knocked down, AQP5 gene expression in both untreated (*p* = 0.007) and hyperosmotic (*p* = 0.038) treatment groups were significantly reduced when compared to the same treatment in shCTR cells (Fig. 3c).

### Effect of TonEBP expression on the in vivo expression of AQP1 and 5

**Figure 4:**
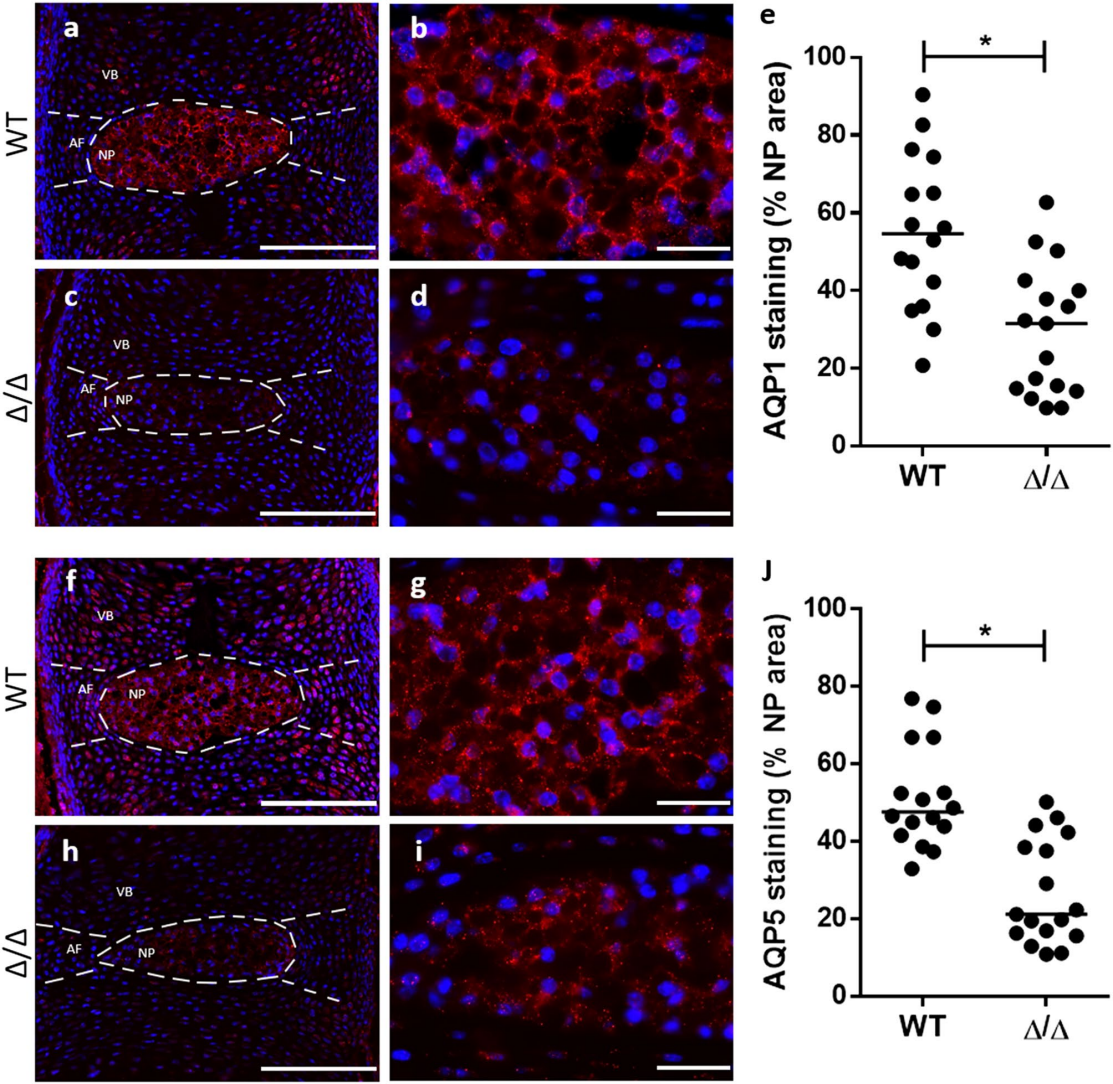
The in vivo expression of AQP1 & 5 in wildtype and TonEBP knockout mice embryos. Fluorescent IHC images showing AQP1 (**a**) and AQP5 (**f**) staining (red) in wildtype (WT) 17.E mouse spines. Increased magnification highlights the localisation of AQP1 (**b**) and AQP5 (**g**) within the nucleus pulposus (NP) region. Fluorescent IHC images showing AQP1 (**c**) and AQP5 (**h**) staining (red) in TonEBP knockout (Δ/Δ) 17.E mouse spines. Increased magnification highlights the localisation of AQP1 (**d**) and AQP5 (**i**) within the nucleus pulposus (NP) region of Δ/Δ mice. Quantification of the percentage NP area containing AQP1 (**e**) and AQP5 (**j**) staining in WT and Δ/Δ mice. The median values of each cohort are represented by the horizontal line in each column. Annulus fibrosus (AF) and vertebral body (VB) regions are depicted (white dashed lines; **a**,**c**,**f**,**h**). Cell nuclei counterstained with DAPI (blue). Scale bar (**a**,**c**,**f**,**h**) = 100 µm. Scale bar (**b**,**d**,**g**,**i**) = 50 µm. Five WT and five Δ/Δ mouse embryos were used for IHC staining, with at least three representative discs per embryo. Statistical significance determined using Kruskal–Wallis test * = *p* ≤ 0.05.

To determine whether AQP1 and AQP5 levels are responsive to TonEBP in vivo, we analysed AQP1 and 5 abundance in discs of TonEBP −/− mice. Since null mice show high perinatal lethality, we chose to stain discs of E17.5 animals. The percentage area of AQP1 fluorescent staining within the NP region of TonEBP −/− mice (Δ/Δ) was significantly reduced compared to AQP1 staining area within the NP region of wildtype (WT) mice (*p* = 0.0007) (Fig. 4a–e). The percentage of AQP5 fluorescent staining within the NP region of TonEBP −/− mice (Δ/Δ) was also significantly reduced compared to AQP5 staining within the NP region of wildtype (WT) mice (*p* < 0.0001) (Fig. 4d–j).

## Discussion

NP cells have adapted to the environment in which they reside. The physiological O_2_ tension^41^, pH^42^, nutrient diffusion^2^, mechanical loading^8^ and osmolality^9^ of the healthy disc allows NP cells to function correctly. When the IVD undergoes degeneration, this environment is altered, exacerbating cellular dysfunction. Previous studies have identified that many AQPs are expressed by NP tissue^29,30,31,39,43,44^. However, only a few studies identify how the expression of AQPs is regulated in the disc. AQP1 and 5 expression are decreased during human IVD degeneration^30^, yet the cause of this, and how both AQPs are regulated in the NP, is unknown. Therefore, for the first time to our knowledge this study has identified that AQP1 and 5 gene expression in NP cells is upregulated by hyperosmolality representative of the normal IVD environment. This could explain why their expression decreases during IVD degeneration, as the osmolality of the NP decreases. This study also determined that the in vitro and in vivo expression of AQP1 and 5 expression in NP cells is TonEBP dependent. As TonEBP is an important transcription factor involved in the overall function of the IVD, the regulation of AQPs by TonEBP may implicate that their function is also important for the health of the IVD.

AQP1 and 5 in human and rat NP cells were upregulated in hyperosmotic conditions that mimic the physiological conditions of the healthy IVD. AQP1 and 5 are also regulated in a similar manner, under similar hyperosmotic conditions, in a range of other tissues and cell types^32–38^, possibly indicating shared mechanisms of regulation. However, hyperosmotic treatment has also led to the reduction of AQP1 and 5 expression in the murine choroid plexus^45^ and hypo-osmotic treatment has led to increased expression of AQP1 in nasal glands^46^, highlighting that the osmotic regulation of AQP expression is also tissue and cell type-specific. 3D hyperosmotic treatment of human NP cells also showed upregulation of AQP1 and 5, along with 2D treatment, indicating that this response was physiologically relevant, as NP cells are known to re-differentiate into an in vivo*-*like state when cultured in 3D.

AQP1 and 5 gene expression in human NP cells was upregulated by hyperosmotic conditions with an osmolality of 425 mOsm/kg, yet the highest upregulation of AQP1 and 5 gene expression in rat NP cells was observed with 525 mOsm/kg treatment. This potentially indicates that there are differences in regulation across species or with age, possibly due to the differences in NP environments and osmotic baselines between adult human and young rat discs. The differences in gene expression may also be a result of the treatment timeframe. AQP1 expression in rat NP cells is significantly upregulated after 8 h treatment, yet after 24 h there is no significant difference compared to baseline levels, and AQP5 was not upregulated at either time point. This may simply be due to AQP regulation by 425mOs/kg occurring before 8 h or after 24 h.

Nevertheless, AQP1 and 5 are regulated by osmolality in a similar manner in human and rodent NP cells. This potentially indicates that osmotic AQP expression is regulated by shared mechanisms and that AQP regulation is important for IVD function across species. Therefore, transitioning to investigate the role of TonEBP on the hyperosmotic regulation of AQP1 and 5 in rats (in vitro) and mice (in vivo), may also reveal the function of TonEBP on such regulation in humans. AQP1 and 5 may enable NP cells to adapt to their hyperosmolar environment, and when expression decreases in degeneration^30^ this may be due to the decrease in the extracellular osmolality, causing further dysfunction of NP cells. This may suggest that AQP1 and 5 expression is decreased, as a consequence of initial degeneration, but also leads to a continuation of the degenerative cascade where cells can no longer adapt to their environment. TonEBP is critical in enabling cells to adapt to a hyperosmotic environment, which is essential for NP cells, as they reside within a hyperosmotic environment during healthy IVD physiology.

This study identified that AQP1 and 5 gene expression, along with their hyperosmotic upregulation, in rat NP cells was significantly reduced when TonEBP expression was knocked down. These results highlight that AQP1 and 5 expression in NP cells is potentially governed by the expression and function of TonEBP. TonEBP has also been shown to regulate the expression of AQP1 and 5 in other tissues^33,34,38^, providing a precedent for results observed in this study. TonEBP expression is also required for the hyperosmotic upregulation of AQP2 in NP cells^39^, therefore AQP1, 2 and 5 may enable the adaptation of NP cells to their hyperosmolar environment along with the classical TonEBP-targeted osmotic response genes.

Along with the classical function in regulating the cell's response to hyperosmotic stress, TonEBP has also been shown to increase the expression of TNF-α^47^, IL-6^48^ and MCP-1^48,49^ under hyperosmotic conditions, all of which are upregulated in IVD degeneration^50–52^. Parallel to the role under hyperosmotic stress, TonEBP is also activated by TNF-α signalling in NP cells, which leads to the upregulation of genes involved in IVD degeneration, rather than the classical osmotic response genes^53^.This highlights that TonEBP may also play a role during IVD degeneration; under hypo-osmotic conditions TonEBP no longer upregulates osmotic response genes, possibly including AQP1, 2 and 5, but rather is activated by TNF-α signalling, intensifying the degenerative cascade^53^.

AQP expression and function has also been implicated in enabling cells to sense osmotic stress via membrane tension changes^54^, interactions with other osmotically activated membrane channels^55,56^, and the control of cell volume regulation^25^, indicating that AQPs may also function upstream of TonEBP to enable cellular adaptation to osmolality changes. It is currently unknown if AQP1 and 5 could function upstream of TonEBP, where AQPs at the membrane could potentially contribute to sensing changes in membrane tension, via modulating the flow of water in response to changes in osmolality^54^. Activated TonEBP could then consequently upregulate AQP1 and 5 further, forming a positive feedback loop ensuring adaptation to the extracellular environment. However, this hypothesis is speculative at present and requires further study. As TonEBP also regulates the expression of NP matrix genes, if AQP1 and 5 expression in NP cells is linked to TonEBP, they may also be linked to matrix production and therefore the fundamental function of NP cells to maintain the integrity of the IVD.

NP expression of AQP1 and 5 was higher in the spines of WT mice compared to TonEBP −/− mice. This suggests that in vivo expression of AQP1 and 5 in the IVD is reliant upon TonEBP expression, strengthening the in vitro regulation also identified in this study. Unfortunately, the in vivo expression of AQP1 and 5 was only able to be investigated during embryonic development due to the lethality of the TonEBP knockdown. Results highlight that during development TonEBP is essential for the maintenance of AQP1 and 5 expression within the NP. However, it is still unknown if TonEBP contributes to in vivo AQP expression in the adult, where many other factors (altered compared to the embryonic environment) could contribute to the physiology of mature NP cells, when compared to the physiology of notochordal cells; the abundant cell type of the NP during development.

## Conclusion

We have previously shown that AQP1 and 5 expression is decreased during human IVD degeneration^30^. This study has identified that AQP1 and 5 are upregulated by hyperosmolality, mimicking the healthy NP, and may explain why expression is decreased during degeneration, when the osmolality is decreased. This suggests AQP1 and 5 may be part of the mechanisms that allow NP cells to adapt to their hyperosmotic environment. TonEBP is an essential transcription factor which enables osmoadaptation, the finding here that the hyperosmotic upregulation of AQP1 and 5 is dependent on TonEBP, implies they both participate in the osmoadaptation process and potential downstream effects on matrix synthesis. During IVD degeneration TonEBP function is uncoupled from the altered osmolality and catabolic genes are upregulated instead of the classical osmotic response genes. As expression of these genes (and AQP1 and 5) is reduced, NP cells can no longer adapt to the degenerate environment and degeneration is exacerbated. Therefore, AQPs may play a role in adapting NP cells to their environment and maintaining the function of NP tissue.

## Materials and methods

### Experimental design

To explore the potential mechanisms for the previously identified decrease in AQP1 and 5 expression in NP tissue during IVD degeneration^30^, AQP1 and 5 expression was determined in human NP cells under osmotic conditions representative of physiological healthy to degenerate IVD. Human NP cells were investigated in 2D (monolayer) and 3D (alginate) culture to determine whether monolayer culture could be used as a model system for osmotic responses, enabling facile manipulation of conditions for future studies. Gene regulation of AQP1 and 5 was investigated in 2D cultured rat NP cells, to determine if responses to hyperosmolality were similar across species as knockdown in vitro studies are optimised for rat cells. Subsequently, TonEBP was knocked down in rat NP cells prior to the application of hyperosmotic stimuli to determine if TonEBP controlled the hyperosmotic regulation of AQP1 and 5 gene expression. To investigate if TonEBP regulated in vivo expression of AQP1 and 5, IHC was performed on the IVDs of WT and TonEBP −/− mouse embryos. All methods were carried out in accordance with relevant guidelines and regulations. Ethical approval was granted from Sheffield Research Ethics Committee (09/H1308/70) for the use of human samples. All animal work was approved by the institutional review board of Thomas Jefferson University.

**Figure 5:**
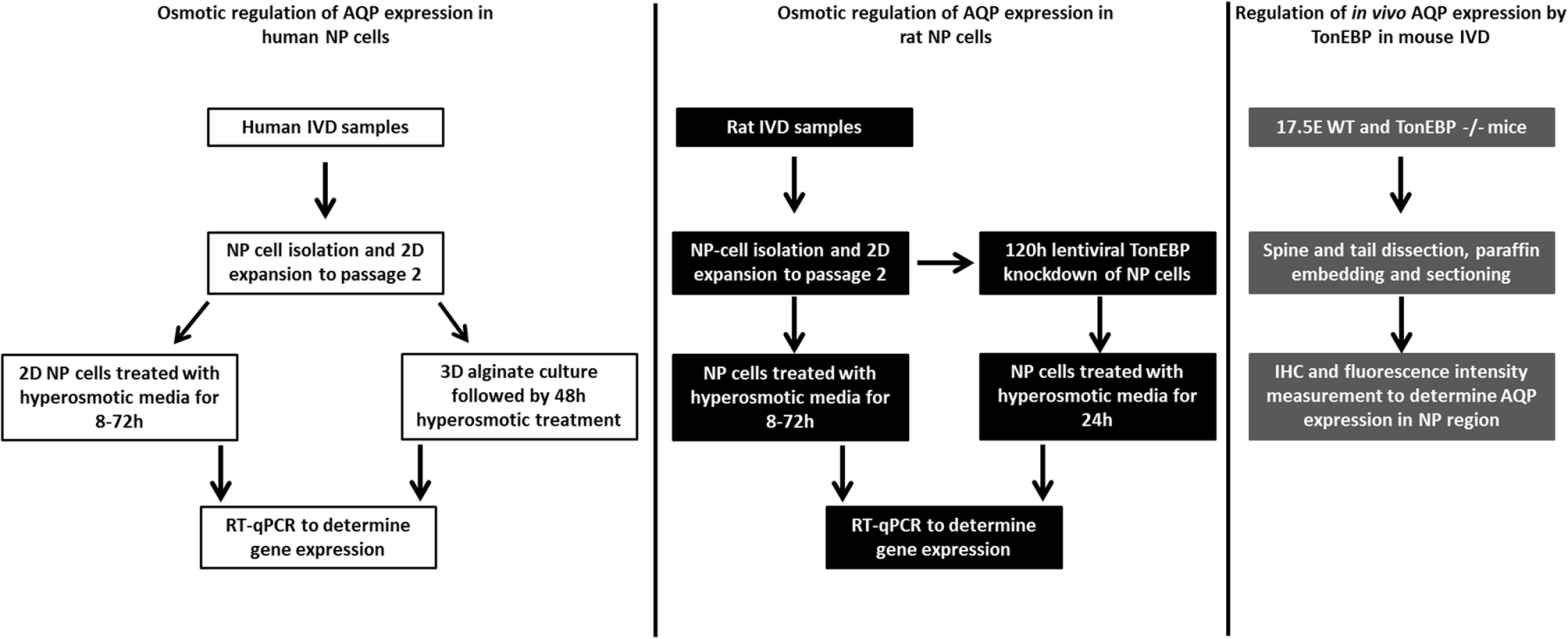
Experimental design to investigate the in vitro osmotic regulation of AQP1 and 5 in NP cells and in vivo regulation of AQP1 and 5 by TonEBP in IVD tissue.

The experimental design is summarised in Fig. 5.

### Human tissue

Human IVD tissue was obtained from patients undergoing microdiscectomy surgery for the treatment of nerve root compression as a result of IVD herniation or post-mortem (PM) examination with informed consent of the patients or relatives. Ethical approval was granted from Sheffield Research Ethics Committee (09/H1308/70).

### Tissue processing

Human IVD tissue was fixed in 10% (v/v) neutral buffered formalin (Leica Microsystems) and embedded into paraffin wax. Following embedding, 4 µm sections were cut and human IVDs histologically graded using haematoxylin and eosin staining methods as described previously^57,58^ to determine severity of IVD degeneration. Samples were separated into individual groups according to grade of degeneration: non-degenerate (grade 0–4), moderately-degenerate (grade 4.1–6.9) and severely-degenerate (grade 7–12).

### Human NP cell extraction and culture

Human NP cells were isolated from surgical and PM tissue (Patient 1: Female, 55 years old, L5/S1, surgical, grade 5; Patient 2: Female, 31 years old, L4/L5, PM, grade 4; Patient 3, Male, 47 years old, L5/S1, surgical, grade 6.5) by digestion with 2 mg/mL collagenase type I (Life Technologies) for 4 h at 37 °C and filtered through a 40 µm cell strainer. Isolated NP cells were expanded in 2D culture using DMEM (Gibco) supplemented with 10% (v/v) FBS, 10,000 U/mL penicillin, 10 mg/mL streptomycin (P/S) (Gibco), 25 µg/mL amphotericin B (Sigma Aldrich), 2 mM l-glutamine (Gibco). Human NP cells at passage 2 were utilised for all experiments.

### Alginate culture

Following expansion up to passage 2, human NP cells were resuspended at a density of 4 × 10^6^ cells/mL in sterile filtered 1.2% (w/v) alginic acid (Sigma-Aldrich) in 0.15 M NaCl. Human NP cell-containing alginate was polymerised by passing through a 20G needle into 0.2 M CaCl_2_ drop-by-drop to produce alginate beads and left for 10 min to fully gel. Newly formed beads were washed with 0.15 M NaCl to remove excess CaCl_2_ and washed twice with DMEM before standard culture media was added. Alginate beads were polymerised and cultured in 24-well plates with 6 beads per well. Alginate beads were cultured in standard conditions for 2 weeks prior to treatment to allow human NP cells to differentiate into an in vivo-like phenotype^59^.

### Rat NP cell extraction and culture

Three Wistar rats (200-250 g) were euthanised with CO_2_ and spinal columns dissected under aseptic conditions. Lumbar IVDs were separated from the spinal column and the NP separated from the AF using a dissecting microscope. NP tissue was partially digested with 0.1% (w/v) collagenase (Sigma-Aldrich) and 10U/mL hyaluronidase (Sigma-Aldrich) for 4-6 h and then maintained in DMEM supplemented with 10% (v/v) FBS and P/S. Rat NP cells migrated out of explant tissue after 1w; when confluent, cells were passaged using trypsin (0.25%) EDTA (1 mM), pooled together and subsequently cultured.

### Hyperosmotic gene regulation of AQPs in human NP cells

Once expanded, human NP cells were seeded into 6-well plates in standard culture media. The osmolality of standard culture media is 325 mOsm/kg and served as the untreated control condition in all experiments; this also mimics the native osmolality of a degenerate disc. Cells were treated with 425 mOsm/kg media for 8, 12, 24, 48 and 72 h to mimic the osmolality of the native, non-degenerate disc in monolayer (2D) culture. To alter the osmolality from 325 to 425 mOsm/kg, 50 mM NaCl was added to media and the osmolality of all solutions was determined using a freezing point osmometer (Model 3320, Advanced Instruments). NP cells in 325 mOsm/kg media at 0 h time point were used as untreated controls. Following treatment, media was aspirated and 1 mL Trizol (Life Technologies) was added to lyse cells. Lysate was collected and 200 μL Chloroform (Sigma-Aldrich) added per mL Trizol (Life Technologies) to extract RNA. RNA was finally resuspended in 14μL RNase-free water (Qiagen, Manchester, UK) before proceeding to cDNA synthesis.

To explore how AQP gene expression was potentially altered by hyperosmotic stimulus in an environment mimicking the in vivo IVD conditions, as compared to 2D culture where NP cells are known to de-differentiate. Human NP cells were resuspended, cultured, and treated following encapsulation into alginate beads. Following 2 weeks of culture in alginate beads to enable re-differentiation, alginate beads were treated (2 beads per well of a 96-well plate) with 325 mOsm/kg (mimicking degenerate conditions), 425 mOsm/kg or 525 mOsm/kg (mimicking healthy conditions), produced by adding 50 mM or 100 mM NaCl added to standard culture media respectively, for 48 h. Human NP cells from 3 patients, each performed with technical triplicates, were used for both 2D and 3D hyperosmotic gene regulation experiments. Following treatment, beads were added to alginate dissolving buffer (55 mM sodium citrate, 30 mM EDTA, 0.15 M NaCl in H_2_O) for 10 min at 37 °C on an orbital shaker before centrifugation at 300 g for 10 min. Supernatant was discarded and pellets resuspended in DMEM containing 0.4 mg/mL collagenase (Sigma-Aldrich) for 10 min at 37 °C on an orbital shaker to degrade extracellular matrix which had been deposited by NP cells during 3D culture. Samples were centrifuged for a further 10 min at 300 g, supernatant removed and 1 mL Trizol (Life Technologies) added to each sample. RNA was extracted using RNeasy mini kit (Qiagen) following the manufacturers guidelines. RNA was finally eluted from RNeasy mini kit columns with 14 μL RNase free water (Qiagen). RNA from both 2D and 3D alginate samples was synthesised into cDNA. qRT-PCR was utilised to identify regulation of gene expression of AQP1 (Hs01033361_m1) and 5 (Hs00387048), employing pre-designed primer/probe mixes (Life Technologies).

### Hyperosmotic gene regulation of AQPs in rat NP cells

Pooled NP cells from 3 rats were seeded at 3 × 10^4^ cells/well in 6-well plates and treated with altered osmolality media (425 mOsm/kg or 525 mOsm/kg) for 8 and 24 h. Experiments were performed in technical triplicates. NP cells cultured in standard media (325 mOsm/kg) at time point 0 h were used as controls. After treatment cells were washed in ice-cold PBS before cell lysis and RNA extraction. RNA was eluted in 20μL RNase-free water. Extracted RNA was then converted to cDNA by adding 20μL RNA to EcoDry premix (Takara) and incubated at 42 °C for 1 h, followed by 10 min at 70 °C. To determine regulation of AQP1 and 5 gene expression under hyperosmotic treatment qRT-PCR was performed for AQP1, 5 and GAPDH.

### TonEBP knockdown in rat NP cells

To investigate the potential role of TonEBP in the hyperosmotic regulation of AQP1 and 5 in rat NP cells, the expression of this transcription factor was knocked down. HEK293T cells were seeded in 10 cm culture plates at 5 × 10^6^ cells/plate in DMEM with 10% (v/v) heat inactivated FBS 24 h prior to transfection. Cells were transfected with 9 μg shCTR (no knockdown control) or shTonEBP (TonEBP knockdown) plasmids along with 6 μg psPAX2 packaging plasmid and 3 μg pMD2.G VSV-G envelope expressing plasmid using Lipofectamine 2000 (Invitrogen). After 6 h transfection media was replaced with fresh DMEM with 10% (v/v) FBS. Lentiviral media was harvested at 48 h and 60 h post transfection and virus was precipitated by adding 7% PEG 6000 solution and storing at 4 °C for at least 12 h. Supernatant/PEG mixture was centrifuged at 1500 g for 30 min at 4 °C to pellet lentiviral particles. Rat NP cells were transduced with fresh media containing viral particles and 8 μg/mL polybrene (Sigma-Aldrich). Knockdown of TonEBP occurred for 5 days. On day 4, hyperosmotic media (425 mOsm/kg) was added to rat NP cells for 24 h of hyperosmotic treatment. Cells were harvested on day 5 of TonEBP knockdown and downstream experiments were performed to assess gene regulation of AQP1 and 5 as described above. Pooled NP cells from 3 rats (in technical triplicates) were used in these experiments.

### TonEBP −/− mice

Nfat5 mutant mice on C57BL/6 background were kindly provided by H. Moo Kwan from the Ulsan National Institute of Science and Technology^60^. Genotyping of Nfat5 mice and embryos was performed by PCR as described previously^60^. The mice were bred socially using aseptic technique with barrier conditions and fed Lab Diet 5010 Laboratory Autoclavable Rodent ad libitum. The litters were of normal size (ranging from 3 to 12) and the embryos were distributed into control and experimental groups based on genotype (wildtype vs −/−). Therefore, it was assumed that the distribution of sex was even between the groups, although this was not objectively determined and cannot be retrospectively confirmed. After sacrifice at E17.5, the spines were dissected and immediately fixed in 4% PFA for 2 days at 4 °C. The spines were subsequently decalcified in 20% EDTA for 3 days at 4 °C and then embedded in paraffin by the Thomas Jefferson University histopathology core facility. All animal procedures were conducted under the guidelines of the Institutional Animal Care and Use Committee of Thomas Jefferson University^21^.

### Immunohistochemistry

Seven-micron sagittal sections were de-paraffinized in histoclear and rehydrated in a series of ethanol solutions (100%-70%). De-paraffinized sections were treated with Proteinase K (1:500) for 10 min at room temperature. The sections were then incubated for 1 h in 5% Normal Goat Serum and subsequently incubated overnight at 4 °C with primary antibody against aquaporin 1 (1:100; abcam, ab168387) and aquaporin 5 (1:100; Millipore, Sigma-Aldrich, A4979). After washing with PBS, sections were incubated for 1 h at room temperature with either Alexa Fluor-594 or Alexa Fluor-647 secondary antibodies (1:700, Jackson ImmunoResearch Lab, Inc.). The sections were washed with PBS before mounting with ProLong Gold Antifade Mountant containing DAPI (Thermo Fisher Scientific, P36934), and visualized by fluorescence microscopy (Axio Imager 2, Zeiss, White Plains, USA) using the 20×/0.5 EC Plan-Neofluar (Zeiss) objective. The stained sections were imaged using X-Cite 120Q Excitation Light Source (Excelitas, Waltham, USA), the AxioCam MRm camera (Zeiss), and Zen2 software (Zeiss). Staining of mouse embryos was performed on five embryos per group (wildtype/NFAT5 null), with at least three representative discs per embryo analysed.

### Digital image analysis

Imaged sections stained by immunohistochemistry were analysed using ImageJ 1.52a^61^ in grayscale. The boundaries of NP cells were digitally traced using the Freehand Tool and the all images were set to the same threshold to create binary images. Designated regions of interest were analysed using the area fraction measurement for each section.

### Statistical analysis

The regulation of AQP1 and 5 gene expression, in both human and rat NP cells, was performed in triplicate on 3 patients/pooled rat cells on different days. Data was found to be non-parametric, therefore Kruskall-Wallis with Dwass-Steel-Critchlow-Fligner post hoc analysis test (Stats Direct) was used to identify significant differences between AQP1 and 5 gene expression. Data from digital image analysis of IHC staining was found to be non-parametric, therefore Kruskall-Wallis with Dwass-Steel-Critchlow-Fligner post hoc analysis test was used to identify significant differences between fluorescent intensities of WT and TonEBP −/− mice, after AQP1 and 5 IHC experiments, to determine putative changes in expression.
